# Myocardial Disease and Long-Distance Space Travel: Solving the Radiation Problem

**DOI:** 10.3389/fcvm.2021.631985

**Published:** 2021-02-12

**Authors:** Manon Meerman, Tom C. L. Bracco Gartner, Jan Willem Buikema, Sean M. Wu, Sailay Siddiqi, Carlijn V. C. Bouten, K. Jane Grande-Allen, Willem J. L. Suyker, Jesper Hjortnaes

**Affiliations:** ^1^Division Heart and Lung, Department of Cardiothoracic Surgery, University Medical Center Utrecht, Utrecht, Netherlands; ^2^Regenerative Medicine Center Utrecht, University Medical Center Utrecht, Utrecht, Netherlands; ^3^Department of Cardiology, University Medical Center Utrecht, Utrecht, Netherlands; ^4^Division of Cardiovascular Medicine, Stanford Cardiovascular Institute, Stanford University School of Medicine, Stanford, CA, United States; ^5^Department of Cardiothoracic Surgery, Radboud University, Nijmegen, Netherlands; ^6^Department of Biomedical Engineering, Technical University Eindhoven, Eindhoven, Netherlands; ^7^Department of Bioengineering, Rice University, Houston, TX, United States; ^8^Division Heart and Lung, Department of Cardiothoracic Surgery, Leiden University Medical Center, Leiden, Netherlands

**Keywords:** radiation-induced cardiovascular disease, cardiovascular system, space radiation, long-distance space travel, experimental studies, countermeasures, HZE ions, heart failure

## Abstract

Radiation-induced cardiovascular disease is a well-known complication of radiation exposure. Over the last few years, planning for deep space missions has increased interest in the effects of space radiation on the cardiovascular system, as an increasing number of astronauts will be exposed to space radiation for longer periods of time. Research has shown that exposure to different types of particles found in space radiation can lead to the development of diverse cardiovascular disease via fibrotic myocardial remodeling, accelerated atherosclerosis and microvascular damage. Several underlying mechanisms for radiation-induced cardiovascular disease have been identified, but many aspects of the pathophysiology remain unclear. Existing pharmacological compounds have been evaluated to protect the cardiovascular system from space radiation-induced damage, but currently no radioprotective compounds have been approved. This review critically analyzes the effects of space radiation on the cardiovascular system, the underlying mechanisms and potential countermeasures to space radiation-induced cardiovascular disease.

## Key points

- Exposure to components of space radiation beyond the low Earth Orbit can have damaging effects on the cardiovascular system, including myocardial remodeling and fibrosis and (micro)vascular damage;
- Several mechanisms of space-radiation induced CVD have been elucidated through experimental studies, such as endothelial dysfunction, increased cellular apoptosis, increased oxidative stress, induction of inflammation and decreased DNA methylation;
- To date, there are no effective measurements to protect astronauts that travel beyond the low Earth Orbit from this damaging type of radiation, and more research should be aimed at finding new methods of protection;
- Current data on this topic is derived from experimental animal or cell culture studies that have significant limitations. Future research should focus on incorporating new techniques, such as the “heart-on-a-chip.”

## Introduction

Since mankind first set foot on the Moon in 1969, the National Aeronautics and Space Administration (NASA) and other space agencies have been working together to expand human space travel into deep space, with the ultimate goal of landing on Mars (1). Consequently, more astronauts will face the serious health risks associated with traveling into deep space. Determining the risks faced by these future space explorers is crucial. Currently, exposure to space radiation is considered one of the most important limiting factors for long-distance space travel (2). Space radiation exposure is linked to the development of cancer and diseases of the central nervous system (CNS) (3). However, over the last few years, there has been growing concern about the effects of space radiation on the cardiovascular system (CVS). Therefore, further research into the effects of space radiation on the development of cardiovascular disease is critical in order to understand and predict the effects of long distance space travel. The aims of this review are to summarize the current knowledge on the effects of space radiation on the cardiovascular system and to discuss potential countermeasures to the development of space radiation-induced cardiovascular disease.

### The Space Environment

#### Space Radiation

In space, astronauts encounter space radiation consisting of galactic cosmic rays (GCR) and solar particle events (SPE) (4, 5). GCR originate outside our solar system and can interact with the Earth's atmosphere, producing showers of secondary particles (5, 6). These rays are mainly composed of high-energy protons (^1^H), together with alpha particles (helium nuclei, ^2+^He), minimal-hazard electrons and positrons and HZE ions [high (H) charge (Z) and energy (E) ions] (7, 8). HZE ions include all nuclei with atomic numbers > 2, of which carbon (^12^C), oxygen (^16^O), magnesium (^24^Mg), silicon (^28^Si) and iron (^56^Fe) are the most prominent (6, 9). Of all the components of GCR, HZE ions are considered the most hazardous to the human body since they can be highly penetrating and can produce secondary particles when they interact with shielding materials like the spacecraft or spacesuit (10). The other component of space radiation, SPE or “proton storms,” are occasionally produced by the Sun and contain large plasma clouds consisting of low- to medium-energy protons, which mostly contain less than a few hundred GeV of kinetic energy (4, 6, 7, 11). Proton exposure doses due to SPE can occur at doses up to 1.5 Gy/h, while GCR exposure occurs at lower dose rates (1.3 mGy/day) (9). The doses that humans are exposed to in space are significantly higher than the radiation exposure humans encounter on Earth, which is estimated to be around 3.1 mSv per year (5).

Radiation can cause damage in proteins, RNA, and DNA in two ways, either directly by direct energy absorption or indirectly via the production of reactive oxygen species (ROS) from the radiolysis of water molecules (4, 8). The amount of biological damage caused by radiation exposure does not only depend on the dose, but also on the type of radiation the target is exposed to. Different types of radiation can be distinguished by the amount of energy transferred to the target material as an ionizing particle passes through, called linear energy transfer (LET). In general, the higher the mass of the ionizing particle, the higher the LET. While the human body is normally exposed to low LET radiation (x-, β- or γ-radiation) on Earth, in space it encounters high LET radiation in the form of HZE ions (3, 12). Two characteristics of high LET radiation make it more hazardous to the human body than low LET radiation. First, radiation types with higher LET produce ion and radical clusters that are close together. Consequently, when a beam of high LET radiation passes through strands of DNA, it typically causes more biological damage than low LET radiation since it induces genomic lesions densely packed around the track of the radiation beam. This is called “clustered DNA damage” (4, 12). These DNA lesions include single- and double-strand breaks, interstrand crosslinks and base modifications. If not repaired, these lesions can result in mutations, chromosome exchanges, carcinogenesis and apoptotic cell death (4, 13). Second, because of the highly ionizing and penetrating capacity of high LET radiation, much lower magnitude physical doses are needed to induce these effects compared to low LET radiation (13). Because the HZE-component of space radiation is more important in deep space beyond the low Earth Orbit (LEO), studying the cardiovascular risks of exposure to this type of radiation is important in light of the current vision to send humans to the Moon and eventually Mars (1, 10).

### Radiation-Induced Cardiovascular Disease (RICVD)

According to a fundamental law in radiobiology (“*Law of Bergonie and Tribondeau*,” 1906), the adult heart has historically been considered a relatively radioresistant organ because of the low proliferation rates of cardiomyocytes (~1% annually) (14). However, research has shown that this perception is not true and that instead the cardiovascular system is indeed very sensitive to radiation (13). Exposure to various types of radiation as described above therefore can lead to radiation-induced cardiovascular disease (RICVD), involving the development of new cardiovascular disease (CVD) or the exacerbation of existing CVD (15). RICVD can either develop within weeks as an acute complication of radiation exposure, mostly being acute pericarditis (12, 16–18), but it can also develop over a longer period of time as chronic RICVD (16, 17). Chronic RICVD is generally progressive and involves multiple disorders of the heart and vasculature, such as myocardial remodeling and fibrosis, accelerated development of atherosclerosis, cardiomyopathies, valve abnormalities, arrhythmias and conduction disorders (12, 13, 16, 17, 19). Retrospective observational studies show that these effects can develop over more than 10–15 years after exposure (13, 16, 17).

RICVD is a well-known complication of radiation therapy in patients treated for thoracic malignancies, such as malignant lymphomas (Hodgkin or Non-Hodgkin lymphoma) or breast and lung cancer (18, 20–23). Radiotherapy treatment for these kinds of malignancies involves exposure of the heart and thoracic vessels (aorta, carotid and coronary arteries) to incidental doses of low LET radiation, which may cause RICVD (21). For instance, a study on Hodgkin's Lymphoma (HL) survivors showed a 3- to 5-fold higher incidence of several types of CVD compared to the general population. They also showed that ~66–80% of heart disease in the HL population was due to radiation exposure during radiotherapy (20). RICVD after breast and lung cancer treatment has also been intensively studied (22, 23). Fortunately, cardiac complications after radiotherapy are currently less common due to modifications in radiotherapy techniques (18).

Besides being observed in patients treated for cancer, RICVD has also been detected in other groups with high exposure to radiation, such as Japanese atomic bomb survivors or occupationally exposed groups, such as the Mayak workers, Chernobyl emergency workers and radiologists before the 1950s (24–26). Cardiovascular morbidity and mortality in >86,000 Japanese atomic bomb survivors, who received radiation doses of 0–4 Gy, have been studied in the Hiroshima-Nagasaki Life Span Study (LSS). This study showed a significantly increased risk for heart disease such as myocardial infarction and an increase in cardiovascular disease risk of 14% per Gy exposure (18, 25, 27).

Taken together, data from these studies demonstrate the development of RICVD in groups with excess exposure to radiation. Yet, care should be taken to extrapolate the data from these groups to astronauts, a highly unique cohort. As discussed above, space radiation is significantly different from radiation encountered on Earth in terms of radiation quality and dose rates. Moreover, cancer patients are generally less healthy than astronauts before exposure, giving rise to potential confounders that might influence the risk of CVD determined in the studies mentioned above (10). Another important consideration is the fact that most astronauts have traveled into space within LEO, while the only astronauts that currently have explored space beyond LEO are the astronauts from the Apollo program. Altogether, it remains difficult to estimate the exact risks astronauts will face during future deep space exploration.

With NASA's current plans to expand human space exploration, more humans will be exposed to the space environment beyond LEO in the near future. A study by Delp et al. reported a 4–5 times higher risk of CVD in Apollo astronauts compared to astronauts who never traveled beyond LEO (28). However, Elgart et al. showed no increased risk of CVD in this population (29). Nonetheless, the group size is limited and these studies therefore both have significant statistical limitations. These conflicting results, combined with the fact that space radiation is currently considered the greatest limiting factor for long-distance space travel (30), emphasize the need for further research into the occurrence of RICVD in astronauts that travel beyond LEO. To date, several experimental studies using different types of animal models have investigated the effects of space radiation on the CVS. The data from these studies will be summarized in the following paragraphs.

## Space radiation-induced cardiovascular disease

### Cardiac Alterations

Myocardial remodeling is a key underlying factor in heart failure; this pathological remodeling includes damage to cardiomyocytes and myocardial fibrosis (31). Myocardial fibrosis is a complicated process which leads to the accumulation of extracellular matrix (ECM) in the myocardium, resulting in a stiffened heart muscle (31). In RICVD, myocardial remodeling and fibrosis are important underlying mechanisms (18). Therefore, these changes may also influence RICVD after exposure to HZE ions in space radiation. To our knowledge, several experimental studies using animal models showed signs of the development of myocardial remodeling or fibrosis after exposure to HZE ions such as protons or ^56^Fe ions, but not after exposure to ^16^O ions or γ-radiation (32–35) (Table 1).

**Table 1:** Overview of experimental animal studies on the effects of space radiation on the CVS.

**Radiation dose (Gy); exposure type**	**Animal model**	**Results**	**Ref.**
**HEART**			
**HZE ions**			
Iron (^56^Fe)			
0.15 Gy, 1 GeV/n; WBE	Male C57Bl/6NT mice, aged 8-10 months	Early systolic and diastolic decompensation (after 1 month) and cardiac hypertrophy (after 3 months), as indicated by an increase in LV posterior wall thickness (PWth), LV end-diastolic pressure (LVEDP), dP/dt_min_, ejection fraction % (EF%) and NFATc4 activity.	(32)
0.5 Gy, 600 MeV/n; WBE	Male C57BL/6 mice, aged 10 weeks	Myocardial remodeling, as indicated by increased collagen deposition and α-SMC actin levels.	(35)
Oxygen (^16^O)			
0.1–1.0 Gy, 600 MeV/n; WBE	Male C57Bl/6J mice, aged 6 months	Myocardial remodeling in the LV, as indicated by dose-dependent increases in the 75 kDa type III collagen cleavage product and increased α-SMC actin levels. No development of cardiac fibrosis.	(34)
**Protons (**^**1**^**H)**			
0.5 Gy, 1 GeV; WBE	Male C57Bl/6NT mice, aged 8–10 months	Cardiac hypertrophy (after 3 months) as indicated by an increase in LV posterior wall thickness (PWth), LV end-diastolic pressure (LVEDP), dP/dt_min_, ejection fraction % (EF%) and NFATc4 activity.	(32)
1 Gy, 1 GeV; WBE	Male C57Bl/6J mice, aged 6 months	Decreased LV α-SMC actin levels	(34)
**γ-radiation**			
Continuous exposure to γ-radiation, 21 days: 0.01 cGy/h, cumulative dose 0.04 Gy; WBE	Female C67Bl/6J mice, aged 6 months	No changes suggestive of myocardial remodeling and fibrosis	(36)
Single exposure; 1.0 and 3.0 Gy; WBE	Male C57Bl/6J mice, aged 6 months	Myocardial remodeling in the LV, as indicated by an increase in the 75 kDa type III collagen cleave product and increased α-SMC actin levels. No development of cardiac fibrosis.	(34)
**Consecutive exposure of different ions**			
0.15 Gy, 1 GeV/n ^56^Fe + 3 × 0.17 Gy, 1 GeV ^1^H; WBE	Male C57Bl/6NT mice, aged 8–10 months	Cardiac hypertrophy and diastolic dysfunction (after 1 month) and increased cardiac fibrosis (after 3 months), as indicated by increased LVEDP and NFATc4 activity.	(33)
0.1 Gy, 150 MeV ^1^H + 0.5 Gy, 600 MeV/n ^56^Fe; WBE	Male C57Bl/6J mice, aged 10 weeks	No changes suggestive of myocardial remodeling	(35)
**VASCULATURE**			
**HZE ions**			
Iron (^56^Fe)			
0.1–0.2 Gy; targeted exposure of the orbital region	Female B6CF1 mice, aged 4 months	Degenerative changes in coronary arteries: smooth muscle degeneration with fibrosis and ECM accumulation in the tunica media.	(37)
2.0 and 5.0 Gy, 600 MeV/n; targeted exposure of the upper aortic tree	Male apoE^−/−^ mice, aged 10 weeks	Accelerated development of atherosclerosis: increased atherosclerotic areas (especially at the aortic root), larger necrotic cores and thickening of the carotid intima.	(38)
1.0 Gy, 1 GeV/n; targeted exposure of the aorta	Male Wistar rats, aged 3–4 months	Increased aortic stiffness and chronic vascular dysfunction.	(39)
**γ-radiation**			
1.0 and 3.0 Gy; WBE	Male C57BL/6 J mice, aged 6 months	Increased collagen deposition in abdominal aorta.	(34)

*α-SMC actin, alpha smooth muscle cell actin; ECM, extracellular matrix; Gy, Gray; LV, left ventricle; LVEDP, left ventricle end-diastolic pressure; NFATc4, nuclear factor of activated T-cells cytoplasmic 4, a marker for cardiac hypertrophy signaling; WBE, whole body exposure*.

Several murine models have demonstrated cardiac remodeling following radiation. Yan et al. first demonstrated the development of progressive cardiac hypertrophy up to 3 months in proton-irradiated hearts (0.5 Gy, from C57Bl/6NT mice), as indicated by an increase in left ventricular (LV) posterior wall thickness (PWth), decreased LV end-diastolic pressure (LVEDP), increased ejection fraction % (EF%) and increased minimum pressure change in the ventricle during the cardiac cycle (expressed as dP/dt_min_). The development of cardiac hypertrophy was confirmed by the increased activity of NFATc4, a marker for cardiac hypertrophy signaling (32). These changes were also observed in mice exposed to ^56^Fe ions (0.15 Gy, 1 GeV/n), but these hearts decompensated earlier and developed systolic and diastolic dysfunction after 1 month, suggesting a stronger effect of ^56^Fe ions compared to protons (32). Another study further confirmed myocardial remodeling of the murine heart induced by exposure to ^56^Fe ions (0.5 Gy, 600 MeV/n) (35). Two common features of myocardial remodeling were both observed in the irradiated hearts, namely increased collagen deposition and increased numbers of myofibroblasts, indicated by higher α-SMC actin levels (35). However, these changes did not occur if these hearts were primed with low dose protons (0.1 Gy, 150 MeV), suggesting a potential protective effect of protons on the heart (35). The most recent study on the effects of exposure to ^56^Fe ions and protons on the murine heart showed that irradiation with a single low dose of ^56^Fe ions (0.15 Gy, 1 GeV/n), followed by 3 doses of protons (0.17 Gy, 1 GeV), caused early hemodynamic alterations of cardiac function 1 month post-exposure (33). These alterations progressed into the development of cardiac fibrosis after 3 months, suggesting synergistic effects of ^56^Fe ion and proton exposure on the cardiovascular system (33).

Yet, some studies have shown that not all particles found in space radiation have fibrotic effects on the myocardium. Seawright et al. reported no changes suggestive of myocardial remodeling and fibrosis in the hearts of C57Bl/6J mice exposed to a continuous low dose of γ-radiation (0.01 cGy/h, cumulative dose of 0.04 Gy), as indicated by a lack of change in α-SMC actin levels, collagen type III content or total collagen composition (36). Another recent publication from the same group showed no effects of exposure to ^16^O ions (0.1–1.0 Gy, 600 MeV/n) or γ-radiation (0.5–3 Gy) on the development of myocardial fibrosis (34). However, they did identify some signs of myocardial remodeling in the left ventricle in response to ^16^O- or γ-radiation, as shown by an increase of a 75-kDa cleave product of type III collagen and α-SMC actin levels (34). In the same study, proton irradiation also showed decreased α-SMC actin levels, again suggestive of a protective effect of protons on myocardial remodeling (34).

From these studies, we can conclude that there is definitely an effect of exposure to ^56^Fe ions, the most prominent heavy ion found in space radiation, on myocardial remodeling, hypertrophy and fibrosis in mice. However, the exact effects of proton irradiation are still unknown, since different doses (0.5 vs. 0.1 Gy) of protons seem to have different effects on the myocardium (32, 34, 35). The potential interplay between different particles found in space radiation is also of concern.

### Vascular Alterations

#### Atherosclerosis

Atherosclerosis is an important topic in RICVD since it has been shown that in groups exposed to higher doses of radiation than the general population, there was a significantly higher prevalence of myocardial infarction (MI) caused by atherosclerosis of the coronary arteries (18, 24–27, 38). Yu et al. reported accelerated development of atherosclerosis after exposure of different areas of the aorta of apolipoprotein E-deficient (apoE^−/−^) mice to 2–5 Gy of ^56^Fe ions (38). They observed increased atherosclerotic areas in these targeted regions of the aorta, whereas no changes were observed in the non-targeted areas, demonstrating a local, non-systemic effect of ^56^Fe ion irradiation on the murine aorta. This effect seemed to be the greatest at the aortic root, demonstrating that this site may be the most sensitive to this type of radiation. The lesions also showed larger necrotic cores, which is associated with instability of plaques and higher risk of thrombogenic complications such as MI or ischemic stroke (40). The authors also detected thickening of the carotid intima after exposure to ^56^Fe ions, indicating that injury to the arterial wall indeed occurred (38). These findings indicate that exposing the cardiovascular system to one of the most prominent components of space radiation, ^56^Fe ions, may cause significant development of atherosclerosis and its associated complications.

#### Microvascular Damage

Arrhythmias and conduction disorders after radiation exposure are not as well studied as other types of RICVD, but some studies suggest that these conditions develop due to microvascular damage. Microvascular damage might cause these conditions as a result of direct damage to the sinoatrial (SA) of atrioventricular (AV) nodes or to cardiomyocyte conduction abnormalities (41). Yang et al. observed degenerative changes in coronary arteries from mice after local irradiation with 0.1–0.2 Gy of ^56^Fe ions. These changes involved smooth muscle degeneration with fibrosis and accumulation of extracellular matrix in the tunica media (37). In a study by Soucy et al., exposure of rat aortas to 1 Gy of ^56^Fe ions led to a significant increase in aortic stiffness and the development of chronic vascular dysfunction by xanthine oxidase (XO)-dependent ROS production and nitroso-redox imbalance, of which the latter has been linked to the development of heart failure (39, 42). Last, a recent study demonstrated a small but significant increase in collagen deposition in the abdominal aorta of C57Bl/6J mice after exposure to γ-rays (1 and 3 Gy) (34). Data from these studies suggest the role of microvascular damage and vascular dysfunction as a cause for the development of CVD after space radiation exposure.

### Biology of Space Radiation-Induced CVD

**Figure 1:**
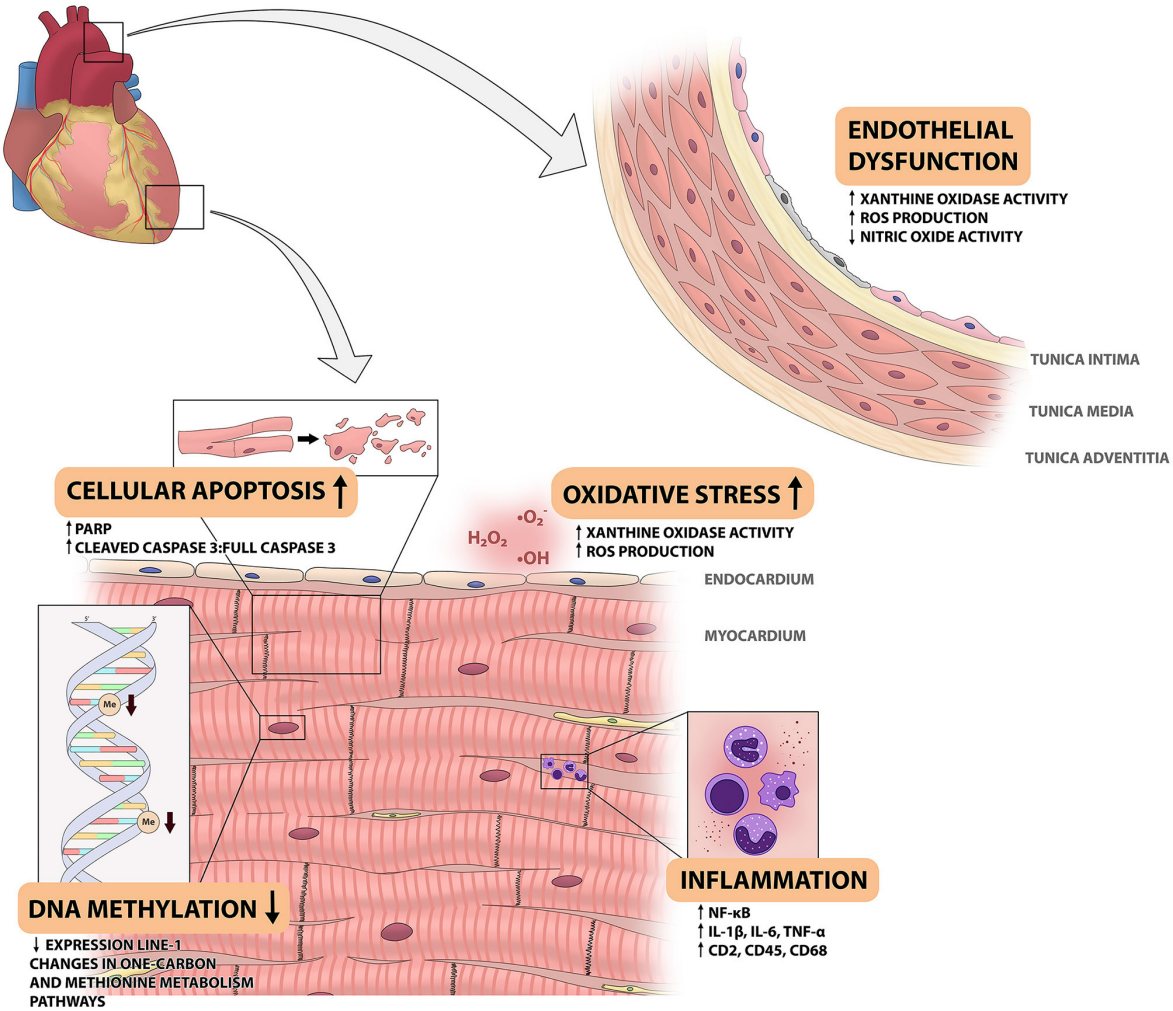
Overview of space radiation-induced changes in the cardiovascular system. This figure shows the changes that have been observed in the cardiovascular system of animal models that are exposed to different components of space radiation. Space radiation has been shown to cause endothelial dysfunction in the aortic wall, as shown on the right. In the myocardium, exposure to space radiation has several effects. It leads to influx of immune cells and increased NF-kB activity, increased reactive oxygen species (ROS) production and xanthine oxidase (XO) activity, increased cellular apoptosis and decreased DNA methylation levels. IL, interleukin; ROS, reactive oxygen species; PARP, poly adenosine diphosphate-ribose polymerase.

To understand how space radiation affects the CVS and to define potential countermeasures, it is important to unravel its underlying mechanisms. An investigation into potential mechanisms is a fairly limited endeavor, as a broad sense of the pathophysiology of space radiation-induced CVD remains to be elucidated. The current knowledge gained from experimental animal studies on the potential mechanisms, will be discussed below with the results summarized in Figure 1.

#### Endothelial Dysfunction

The functional capacity of the endothelium is believed to play a central role in the development of RICVD, partly since endothelial dysfunction is associated with a proinflammatory and profibrogenic environment (12, 35). For this reason, the majority of previous radiation studies (cell culture and animal models) have focused on endothelial cells. Lauk et al. first showed that in cardiac epithelial cells from irradiated Wistar and Sprague-Dawley rats, loss of alkaline phosphatase (AP) activity, a marker of functional epithelium, occurred before the development of myocardial generation and symptoms of heart disease (43). Such findings have motivated further research into the effects of space radiation on the endothelium. For example, Soucy et al. irradiated targeted segments of rat aortas with 1 Gy (1 GeV/n) of ^56^Fe ions (39). The irradiated regions showed signs of dysfunctional endothelium, as measured by a diminished endothelium-dependent relaxation, compared to the non-irradiated parts. This development was linked to increased xanthine oxidase (XO) activity and ROS production and decreased nitric oxide (NO) production (39). Another feature of endothelial dysfunction is an imbalance in the thrombomodulin (TM) system. TM is a transmembrane glycoprotein found in endothelial cells and has anti-fibrinogenic and anti-inflammatory properties. Expression of TM can be used to assess endothelium functionality, since TM is cleaved off the endothelial cell surface during the development of endothelial cell dysfunction. Ramadan et al. found increased cardiac TM levels after irradiation with 0.5 Gy of ^56^Fe ions (35). Despite the link between the TM-system and endothelial dysfunction, identifying the precise role of TM in RICVD will require more intensive study.

#### Cellular Apoptosis and Senescence

Irradiation of cardiac tissue has been previously shown to cause apoptotic cell death of various cardiovascular cell types, including cardiomyocytes, cardiac myofibroblasts, conducting and vascular tissues (16). Apoptosis has been associated with myocardial damage and is therefore an important subject to study in research on the effects of space radiation on the CVS (19). In a study conducted with 300 MeV/n ^28^Si ions, irradiated murine hearts showed higher levels of apoptotic cell death and inflammation up to 6 months after exposure, as measured by increased levels of cleaved poly(adenosine diphosphate-ribose) polymerase (PARP), a marker for apoptotic cell death (19). In other reports, a study using ^56^Fe ions showed increased levels of apoptotic cell markers after radiation exposure, as shown by an increase in the ratio of cleaved caspase 3 to the full caspase 3 protein (35). Even though ^28^Si and ^56^Fe ions have been shown to cause significant apoptosis in murine hearts, these effects were not observed after exposure to ^16^O ions (34). It is therefore plausible to conclude that the occurrence of apoptosis after exposure to heavy ions is radiation type-specific and does not occur after exposure to every heavy ion found in space.

#### Inflammation

The immune system is involved in atherogenesis and myocardial remodeling and fibrosis, with important roles for macrophages, lymphocytes, mast cells and pro-inflammatory cytokines (10, 31). How the immune system contributes to the development of space radiation-induced CVD is therefore an important question. Tungjai et al. demonstrated the induction of a chronic inflammatory state in the hearts of CBA/CaJ mice up to 6 months after exposure to ^28^Si ions, as indicated by persistently increased levels of NF-κB and associated pro-inflammatory cytokines such as IL-1β, IL-6, and TNF-α (19). Chronic activation of NF-κB has been shown to affect the cardiovascular system negatively by stimulating the production of pro-inflammatory cytokines, which can lead to chronic inflammation, cell death and heart failure (44). After exposure to ^56^Fe [0.5 Gy, 600 MeV/n (34, 35) or 15 cGy, 1 GeV/n (45)] and ^16^O ions (0.1–1.0 Gy, 600 MeV/n), C57Bl/6J mice demonstrated increased cardiac levels of mast cell tryptase, the T-lymphocyte marker CD2 and the monocyte/macrophage marker CD68. Conversely, an increase in the leukocyte marker CD45 was only observed after ^16^O radiation (34, 35). Besides, cardiomyocytes isolated from ^56^Fe irradiated mice (15 cGy, 1 GeV/n) showed increased activity of inflammatory and free-radical scavenging pathways, as demonstrated by time-dependent changes in gene expression. Taken together, these findings suggest that exposure of the murine heart to heavy ions found in space radiation can lead to induction of a chronic inflammatory state, which is associated with decreased cardiac function caused by oxidative stress and apoptotic cell death, induced by the release of pro-inflammatory cytokines, superoxide, nitric oxide, and other signaling molecules (36, 45).

#### Oxidative Stress

Radiation exposure can damage the cardiovascular system via oxidative stress in multiple ways, of which an excellent summary can be found in Takahashi et al. (46). Since cardiomyocyte membranes are very rich in phospholipids that are sensitive to ROS, oxidative stress is an important mechanism in radiation's damaging effects (16). Several studies in which cultured cardiomyocytes were exposed to free radicals, as produced during exposure to radiation, have shown depressed contractile function, structural abnormalities, enhanced levels of phospholipid peroxidation, impaired energy production, and increased resting tension (16, 47). Furthermore, other physical stimuli in space—in addition to radiation—lead to oxidative stress, resulting in upregulated expression of oxidative enzymes and downregulated expression of anti-oxidative enzymes (46). A recent study identified one gene, FYN, that is upregulated after exposure to oxidative stress caused by space radiation and that reduces ROS levels (3). This pathway might function as an intrinsic mechanism to protect the cardiovascular system against the damaging effects of ROS. However, this putative mechanism has thus far only been observed in murine cardiomyocytes and human endothelial cells (HUVECs), so it would be interesting to see if this mechanism also occurs in whole organisms, such as mice. Overall, the exact role of oxidative stress requires further attention since there are currently limited studies who describe its effects on pathogenesis of space radiation-induced CVD.

#### DNA Methylation

There is increasing interest in the role of DNA methylation, an epigenetic mechanism with an important role in cellular homeostasis, in the pathogenesis of CVD (48, 49). Emerging evidence suggests that space radiation, in part, exerts its effects on the cardiovascular system through alterations in DNA methylation, especially in repetitive elements of the genome (49, 50). In studies on DNA methylation, retrotransposon LINE-1 is often used as a marker for global DNA methylation levels since it is the most prevalent repetitive element in mammalian genomes (51).

Animal studies have shown that exposure of the murine heart to ^56^Fe ions, ^16^O ions, and protons leads to hypomethylation and decreased expression of repetitive elements such as LINE-1 (49, 50). Koturbash et al. also observed changes in components of the one-carbon and methionine metabolism pathway. These pathways are involved in DNA methylation via the synthesis of the methyl groups used in the methylation process, namely S-adenosylmethionine (SAM) (52). These changes in the methionine cycle have been suggested to impair DNA methylation, therefore intensifying the primary effects of space radiation on DNA methylation. Lastly, altered DNA methylation may serve as an early biomarker for space radiation exposure, since changes in DNA methylation are observed months after the initial exposure (36, 49, 50). This might give rise to personalized treatment based on the level of altered DNA methylation after exposure. However, the exact link between the level of exposure to space radiation, the amount of altered DNA methylation and the development of CVD has not been established yet.

### Potential Countermeasures and Protection Methods

Since space radiation exposure is considered to be the most important limiting factor for long-distance space travel, new methods of radiation protection or scavenging have to be developed in order to guarantee astronauts' safety during future space missions. There are two possible approaches for radiation protection in space. One of them is providing a physical barrier between the astronaut and space radiation by means of shielding materials. Another is the administration of radioprotective pharmacological agents. Both approaches will be discussed below.

#### Shielding

Currently, the only protection method against space radiation is by the use of radiation shielding since increasing the distance from the radiation source is impossible and the amount of time exposed to space radiation cannot be limited any further if the goal is to extend space missions into deep space (2, 6). However, radiation shielding is not easily achieved in space. Current shielding methods are sufficient in protecting against space radiation inside the LEO, but are not suitable for the space environment beyond the LEO (6). The main problem regarding radiation shielding in deep space is the production of secondary particles when HZE ions encounter shielding materials such as the spacecraft (10). Research has shown that although shielding against SPE could be effective, it is currently not yet possible to shield against GCR effectively. There have been speculations that active shielding, which comprises the generation of electromagnetic fields to avert cosmic rays, might be interesting in protecting against GCR, but this technique is not applicable in practice yet (6).

#### Pharmacological Protection

Because of the lack of adequate shielding possibilities, there has been increasing interest in the use of pharmacological compounds to limit the damaging effects of space radiation. Generally, such compounds can be divided into three categories, including radioprotectors (which decrease or prevent tissue damage before exposure), radiomodulators (which increase baseline resistance to radiation exposure) and radiomitigators (which limit or prevent tissue damage after exposure) (53). Over the past few years, several candidate drugs and antioxidants with such properties have been examined in the context of protection against space radiation exposure.

##### Drugs

Since the effects of space radiation on the human cardiovascular system and its mechanisms have not been fully elucidated, very few compounds have been evaluated as of yet in a simulated space radiation environment. The angiotensin converting enzyme (ACE) inhibitor captopril seems to be able to reduce radiation-induced cardiopulmonary complications in animal models, but data are limited (53). However, it has been argued that ACE inhibitors would make poor prophylactic agents in the astronaut population because of severe side-effects in the space environment, such as decreased renal perfusion and angioedema (53). The use of the xanthine-derivative pentoxifylline combined with α-tocopherol showed beneficial effects on myocardial fibrosis and left ventricular function in animal models, but these results have not been reproduced in a model of space radiation yet (54). Statins showed promising results on reducing radiation-induced atherosclerosis, but conflicting results have been published (17). Other compounds that have been evaluated without any success have been discussed elsewhere and are beyond the scope of this review (8, 53). In conclusion, no safe pharmacological compounds are currently identified to use as prophylaxis in astronauts exposed to space radiation, motivating an ongoing search for suitable compounds.

##### Antioxidants

The antioxidant family forms a promising group of potential radioprotectors. As aforementioned, exposure to space radiation is associated with oxidative stress because of the production of ROS in the interaction between HZE ions and water in biological tissues (4, 46). Antioxidants are enzymatic or non-enzymatic substances that limit the amount of ROS in normal tissue by removing these ROS in several steps, thereby preventing the possible damaging interactions between ROS and DNA (47). Thus, antioxidants have been of interest for treatment of CVD for years and might also serve as prophylaxis against space radiation-induced oxidative stress (8, 55). Kennedy et al. showed that combined doses of the antioxidants N-acetyl cysteine (NAC), ascorbic acid (vitamin C), α-lipoic acid (a type of vitamin B), coenzyme Q10, vitamin E succinate, sodium ascorbate and L-selenomethionine (SeM) were able to reduce oxidative stress in cultured cells. They also irradiated rats and mice with ^56^Fe ions (0.5 Gy, 1 GeV/n), γ-rays or protons (both 3 Gy), which led to a significant decrease in total antioxidant status (TAS). After the animals were fed with a diet supplemented with various combinations of the above mentioned antioxidants, TAS increased significantly and even returned to normal pre-radiation exposure levels when combined with the Bowman-Birk Inhibitor Complex (BBIC; a protease inhibitor derived from soy beans) (8). Amifostine, a radioprotective agent that is already being clinically used in cancer patients, also showed cardioprotective effects after single doses of radiation exposure in rats, but currently has too many side-effects to be used by astronauts (56). Furthermore, hydrogen therapy in the form of hydrogen-enriched water or hydrogen gas inhalation could be another way to protect astronauts from oxidative stress since hydrogen showed strong antioxidant properties in several studies. However, data on hydrogen therapy is still limited (57). Next to the administration of exogenous antioxidants, certain diets can also be used to manipulate the endogenous antioxidant balance. Beets, green vegetables, tomatoes and milk- and yeast-derived foods contain certain compounds that can have antioxidant properties as well, as discussed earlier by Hughson et al. (10). Additionally, there has also been some interest in certain diets that have proven to be beneficial to the cardiovascular system, such as calorie-restricted and low-sodium diets (10).

The use of antioxidants in space faces several limitations. The reduction of ROS through antioxidant use in animal models is reportedly accompanied by increased chronic inflammation of the cardiac tissue, which is also known to be associated to the development of CVD (3). It will be important to determine if this also occurs in the human cardiovascular system. Another important limitation, as discussed by Hughson et al., is the possible interaction between antioxidants and the high (100%) oxygen concentrations that astronauts are briefly exposed to during extravehicular activities (10). An increase in inhaled oxygen concentration could lead to decreased antioxidant and radioprotective properties (10). Also, few studies have been conducted regarding the underlying mechanisms of the ROS-reducing capacity of antioxidants after space radiation exposure. Last, implementing the suggested diet changes in astronauts is also challenging since the cardiovascular effects of the space environment are not the only factors to take into consideration. For example, a calorie-restricted diet is not suitable for astronauts since they exercise daily and face bone and muscle atrophy as a result of prolonged microgravity, which requires a specialized diet high in calories as a countermeasure (10).

In conclusion, current data on the efficacy of antioxidants in reducing or preventing space radiation-induced CVD is not sufficient to introduce them as radioprotective agents in astronauts. Even though some studies in animal and cell culture models show promising results, we still face several limitations regarding the implementation of these compounds in practice. However, in the future these compounds may function as radioprotective substances in combination with other radioprotective measures to prevent astronauts from space radiation-induced CVD.

## Concluding remarks

Research on the cardiovascular effects of space radiation has increased significantly over the last few years. For space agencies, this field of research is crucial to estimate the health risks astronauts will face during and after long-distance space travel beyond the LEO. Furthermore, understanding the pathophysiological mechanisms of space radiation-induced CVD should lead to better ways to protect astronauts from these conditions. These results also have implications for life on Earth, as they can contribute to a better understanding of CVD on Earth, with and without radiation exposure.

However, there are several limitations regarding current research on the effects of space radiation on the CVS. First, a well-known complication of exposure of the heart to radiation is damage to the heart valves, which may result in valve stenosis of regurgitation (58). However, none of the studies discussed in this review have focused on the changes in valve structure and function after exposure to components of space radiation. Yu et al. do report increased development of atherosclerosis, especially in the aortic root, which could also affect the aortic valve (AV) (38). Yet, AV structure and function was not included in their analysis. Considering the fact that valvular disease is an important contributor to the global cardiovascular disease burden (59, 60), future studies should also focus on the consequence of space radiation exposure to the heart valves.

Another limitation involves the scarcity of epidemiological data on the incidence of CVD after long-distance space travel in humans, since only the Apollo crew traveled beyond the LEO, and their exposures were quite short. Even though Delp et al. reported a significantly increased risk of CVD in the group of Apollo astronauts (28), their results have been criticized by other researchers because of several limitations in their methods (10, 61). For example, they did not account for confounding factors that might influence the development of CVD in the Apollo astronauts and did not include other space missions and radiation exposures (10). Besides, another study showed no increased risk of CVD in this group (29).

Because of the lack of data in humans, the effects of space radiation are currently most commonly investigated using cultured cell lines and animal models, which both have their individual limitations. Wnorowski et al. recently reported on the effects of microgravity on the structure and function human-induced pluripotent stem cell-derived cardiomyocytes that were cultured in the International Space Station (ISS) (62). However, the biggest disadvantage of such models is the inability to study biological processes in a living, complex organism that is more similar to human beings, which is possible with animal models (63). However, animal models also have their limitations. To study the pathophysiology of atherosclerosis, atherosclerosis-prone mouse models such as the apolipoprotein E-deficient (apoE^−/−^) model have to be used since regular mouse models are relatively resistant to atherosclerosis (64). This susceptibility makes it challenging to translate these results to healthy astronauts who lack any prior cardiovascular risk factors. Furthermore, a major limitation of apoE^−/−^ mice is the lack of thrombogenic complications and plaque rupture (65). Indeed, all of the animal models used in the studies discussed above have limitations. To our knowledge, no studies have been conducted with larger animal models that might better resemble the human cardiovascular system and pathogenesis of atherosclerosis (64, 65). Nonetheless, the discussed studies do show that components of space radiation cause significant damage to the cardiovascular system, which has to be further explored in future studies.

Another important limitation in current space radiation research is the possibility of mimicking the space radiation environment on Earth. The studies discussed above used single ions such as ^56^Fe or ^16^O ions, but in space astronauts will encounter different particles simultaneously or consecutively. In some studies it was already observed that the combined exposure to different particles had different effects on the cardiovascular system (33–35). To gain more precise insights into the cardiovascular effects of exposure to radiation in deep space, new methods must be developed to study the combined exposure to different particles. Additionally, several HZE ions are currently understudied. Even though ^56^Fe ions account for around 20% of the biological damage caused by HZE ions, very few studies have investigated the effects of other ions on the cardiovascular system, such as ^28^Si or ^16^O ions (19, 34). To our knowledge, no studies using magnesium ions (^24^Mg) have been conducted yet, even though this ion is also part of the HZE-component of space radiation. Future research should focus on the combined exposure of different heavy ions found in space radiation beyond the LEO and more attention should be drawn to currently understudied ions such as oxygen, magnesium and silicon ions. The facilities of the NASA Space Radiation Laboratory (New York, USA) and the knowledge and experience of its researchers could be of great value in future research on this topic (66).

Lastly, space radiation is not the only limiting factor for long-distance space travel. To examine the exact changes the CVS undergoes in the space environment, other factors such as prolonged microgravity, hypoxia and disrupted circadian rhythms should also be considered. This is especially important since altered blood flow patterns, oxidative stress and sleep deprivation are all recognized as cardiovascular risk factors, and may therefore add to the increased risk of spaceflight-associated CVD (67). However, the effects of these other space-specific factors are outside of the scope of this review. Research in which exposure to space radiation is combined with these other space-specific factors should yield the most reliable results on the effects of the space environment on the development of CVD. Unfortunately, this type of research has not yet been conducted at the present time.

The abovementioned limitations show that both conventional cell culture platforms and animal models are not suitable for studying the effects of space radiation on the human CVS. Conventional *in vitro* cell culture platforms are not able to mimic the complex and dynamic environment of the CVS, while the CVS of the animal models used in the discussed studies are significantly different from the human CVS, which makes translating these results into humans difficult. A potential interesting alternative approach is the implementation of the “organ-on-a-chip” technology into this field of research. “Organs-on-chips” are able to incorporate different types of human cardiovascular cells in a model that is able to recapitulate the near-physiological environment of the human heart. These models have also been of great interest in the field of drug discovery and screening, which could aid in the development of new protective measures against space radiation exposure (68, 69). Also, these models would enable researchers to study the combinatorial effects of space radiation exposure with other space-specific factors such as hypoxia, which would mimic the space environment more accurately. To our knowledge, no studies into the effects of space radiation on the human CVS have been conducted using “organs-on-chips” yet. However, these models show great promise and future research should focus on implementing such models into their experimental setup.

Despite the abovementioned limitations, data from the experimental studies discussed in this review show that the cardiovascular system is undoubtedly very sensitive to the damaging effects of space radiation. These results emphasize the need for better protective measures as more astronauts will travel into deep space. Yet, no effective compounds have been approved. Since the mechanisms of space radiation-induced CVD are slowly being unraveled, future research should focus on identifying compounds that interfere with these mechanisms. Besides, the potential benefit of antioxidants should be further explored and tested in human models such as the “organ-on-a-chip” in order to translate these results into practice. Before any recommendations can be made regarding the administration of pharmacological compounds or antioxidants to astronauts who will travel beyond the LEO, further research must be performed into the underlying mechanisms and pharmacological characteristics such as the optimal dose, side-effects and possible interactions of these compounds, in order to safely protect our astronauts. In summary, data gained from experimental animal studies show that several components of space radiation, such as HZE ions and protons, can have serious harmful effects on the CVS and therefore can lead to the development of space radiation-induced CVD. Since the rising interest in the effects of space radiation on the CVS in the last few years, few studies on this topic have been published to date, and we might have only seen a small aspect of the effects of space radiation on the CVS. One of the main questions that arises from the plans to expand human space exploration to Mars is whether the risks astronauts face during and after these future space missions outweigh the benefits of long-distance space travel. With current knowledge gained from animal and cell culture studies, it is not yet possible to answer this question. However, we now know that if the human CVS responds to space radiation in any similar way to the murine cardiovascular system, long-distance space travel can lead to several serious types of CVD and therefore affect the astronauts' health tremendously. Thus, future research should be focused on determining the exact effects of space radiation on the CVS, unraveling the underlying pathological mechanisms, and designing countermeasures in order to protect our future space explorers to the fullest extent possible.

## Author Contributions

MM and JH wrote the manuscript. CB, TBG, JB, SW, SS, KG-A, and WS contributed to editing the final version of the manuscript. All authors contributed to the article and approved the submitted version.

## Conflict of Interest

The authors declare that the research was conducted in the absence of any commercial or financial relationships that could be construed as a potential conflict of interest.

## Abbreviations

GCR: galactic cosmic rays
HZE: high charge (Z) and energy (E)
LET: linear energy transfer
LEO: low Earth Orbit
SPE: solar particle events.
